# Novel Application of Nanofluidic Chip Digital PCR for Detection of African Swine Fever Virus

**DOI:** 10.3389/fvets.2020.621840

**Published:** 2021-02-05

**Authors:** Rui Jia, Gaiping Zhang, Hongliang Liu, Yumei Chen, Jingming Zhou, Yankai Liu, Peiyang Ding, Yanwei Wang, Weimin Zang, Aiping Wang

**Affiliations:** ^1^School of Life Sciences, Zhengzhou University, Zhengzhou, China; ^2^Henan Zhongze Biological Engineering Co. LTD, Zhengzhou, China

**Keywords:** African swine fever virus, chip digital PCR, sensitive detection, application, nanofluidic

## Abstract

African swine fever virus (ASFV) gives rise to a grievous transboundary and infectious disease, African swine fever (ASF), which has caused a great economic loss in the swine industry. To prevent and control ASF, once suspicious symptoms have presented, the movement of animal and pork products should be stopped, and then, laboratory testing should be adopted to diagnose ASF. A method for ASFV DNA quantification is presented in this research, which utilizes the next-generation PCR platform, nanofluidic chip digital PCR (cdPCR). The cdPCR detection showed good linearity and repeatability. The limit of detection for cdPCR is 30.1995 copies per reaction, whereas no non-specific amplification curve was found with other swine viruses. In the detection of 69 clinical samples, the cdPCR showed significant consistency [91.30% (63/69)] to the Office International des Epizooties-approved quantitative PCR. Compared with the commercial quantitative PCR kit, the sensitivity of the cdPCR assay was 86.27% (44/50), and the specificity was 94.44% (17/18). The positive coincidence rate of the cdPCR assay was 88% (44/50). The total coincidence rate of the cdPCR and kit was 89.86% (62/69), and the kappa value reached 0.800 (*P* < 0.0001). This is the first time that cdPCR has been applied to detecting ASFV successfully.

## Introduction

African swine fever (ASF) was first reported in Kenya, Eastern Africa, in 1921 and then gradually swept across the globe to this day (1, 2). With the death rate of the swine approaching 100%, ASF is putting downward pressure on the global economy and is a disaster for the pig industry (3, 4). African swine fever virus (ASFV), the pathogen causing ASF, is a large double-stranded DNA virus with an envelope and is the only member of the *Asfivirus* genus in the *Asfarviridae* family (5). The genome size of ASFV is from 170 to 190 kb, so it belongs to the nucleocytoplasmic large DNA viruses (6, 7).

Domestic pigs with ASFV infections have serious clinical manifestations such as acute hemorrhagic fever, dyspnea, serous or mucopurulent conjunctivitis, bloody dysentery, vomiting, among others (8). Currently, there are no effective treatments, and vaccine research is progressing slowly. Once suspicious symptoms of ASF presented, the most valid measurements are to firstly stop all circulation of animals and pork products, for example, *via* animal isolation and traffic restriction (9–11). ASF can then be confirmed by a laboratory test (12).

The laboratory diagnostic approaches of ASF are mainly divided into two groups: one includes isolating the virus, detecting virus antigens and genomic DNA, whereas the other aims at detecting an antibody (12, 13). Polymerase chain reaction (PCR) technique is the most mature molecular method for determining virus genomic DNA/RNA. Conventional PCR (14) and fluorescent quantitative PCR (qPCR) (15) have been applied in testing ASFV. Several PCR technologies have been established to achieve quantitative analysis for the concentration of virus DNA during amplification. Real-time fluorogenic qPCR is the most frequently used form of qPCR, in which concentrations of samples are calculated from initial concentrations of standard sample templates. Currently, although qPCR has been used in ASFV detection studies to measure the virus genomic DNA (16–21), digital PCR is getting increasingly popular because it realizes absolute quantification without reliance on external standards, standard curves, and the cycle within the amplification process that the reporter dye signal exceeds a threshold [cycle threshold (CT) value] (22).

Nanofluidic chip digital PCR (cdPCR), a type of digital PCR supported by QuantStudio 3D (Applied Biosystems, US), adopts a sealed chip that partitions samples into thousands of reaction wells to run independent PCR amplifications. When amplifications are finished, the concentration of the target gene in the original sample is calculated by counting and converting positive wells, which have positive amplification of the viral target gene using the Poisson model correction coefficient (22, 23). Another superiority of cdPCR is the high sensitivity, which makes it a dream platform for studying (24, 25) low-level pathogen detection (26, 27) as well as absolute quantification of viral load (28).

This study focuses on the application of cdPCR, in which ASFV is detected by designing a pair of primers and the minor groove binder (MGB) probe in the portion sequence of the ASFV B646L gene. Applicability of this new ASFV diagnosis methods is evaluated in terms of sensitivity, specificity, and coincidence rate with qPCR approved by the Office International des Epizooties (OIE) and commercial qPCR kits.

## Materials and Methods

### Probe and Primers

We designed a set of the MGB probe [5′-(FAM)-ACTGGGACAACCAAAC-3′-(MGB)], upstream primer (ASFV-For: 5′-ACGTTTCCTCGCAACGGATA-3′) and downstream primer (ASFV-Back: 5′-CGTGTAAACGGCGCCCTCTAA-3′), which aimed at the B646L gene (Genebank: MK128995.1) using PRIMER EXPRESS software (version 1.5, Applied Biosystems, USA). The size of the target gene was approximately 63 bp. Primers and the probe sequences were compared with genes of some various ASFV strains sequences in the GenBank database (Table 1).

**Table 1:** The primers and MGB probe were aligned with 53 ASFV epidemic strains and 5 other swine pathogenes.

**ASFV isolate**	**GeneBank accession number**	**Target sequence**
ASFV/pig/China/CAS19-01/2019	MN172368.1	ACGTTTCCTCGCAACGGATATGACTGGGACAACCAAACACCCTTAGAGGGCGCCGTTTACACG
ASFV/LT14/1490	MK628478.1	ACGTTTCCTCGCAACGGATATGACTGGGACAACCAAACACCCTTAGAGGGCGCCGTTTACACG
CzechRepublic 2017/1	LR722600.1	ACGTTTCCTCGCAACGGATATGACTGGGACAACCAAACACCCTTAGAGGGCGCCGTTTACACG
taibntMoldova2017/1	LR722599.1	ACGTTTCCTCGCAACGGATATGACTGGGACAACCAAACACCCTTAGAGGGCGCCGTTTACACG
ASFV-wbBS01	MK645909.1	ACGTTTCCTCGCAACGGATATGACTGGGACAACCAAACACCCTTAGAGGGCGCCGTTTACACG
Belgium2018/1	LR536725.1	ACGTTTCCTCGCAACGGATATGACTGGGACAACCAAACACCCTTAGAGGGCGCCGTTTACACG
ASFV-SY18	MH713612.1	ACGTTTCCTCGCAACGGATATGACTGGGACAACCAAACACCCTTAGAGGGCGCCGTTTACACG
Georgia 2007/1	NC_044959.1	ACGTTTCCTCGCAACGGATATGACTGGGACAACCAAACACCCTTAGAGGGCGCCGTTTACACG
47/Ss/2008	NC_044955.1	ACGTTTCCTCGCAACGGATATGACTGGGACAACCAAACACC**T**TTAGAGGGCGCCGTTTACACG
ETH/1a	KT795359.1	ACGTTTCCTCGCAACGGATATGACTGGGACAACCAAACACCCTT**G**GAGGGCGCCGTTTACACG
AnhuiXCGQ	MK128995.1	ACGTTTCCTCGCAACGGATATGACTGGGACAACCAAACACCCTTAGAGGGCGCCGTTTACACG
ETH/2a	KT795358.1	ACGTTTCCTCGCAACGGATATGACTGGGACAACCAAACACCCTT**G**GAGGGCGCCGTTTACACG
POL/2015/Podlaskie	MH681419.1	ACGTTTCCTCGCAACGGATATGACTGGGACAACCAAACACCCTTAGAGGGCGCCGTTTACACG
R7	MH025917.1	ACGTTTCCTCGCAACGGATATGACTGGGACAACCAAACACC**T**TT**G**GAGGGCGCCGTTTACACG
ETH/1	KT795354.1	ACGTTTCCTCGCAACGGATATGACTGGGACAACCAAACACCCTTGGAGGGCGCCGTTTACACG
ETH/AA	KT795353.1	ACGTTTCCTCGCAACGGATATGACTGGGACAACCAAACACCCTT**G**GAGGGCGCCGTTTACACG
BA71	NC_044942.1	ACGTTTCCTCGCAACGGATATGACTGGGACAACCAAACACC**T**TTAGAGGGCGCCGTTTACACG
Ken05/Tk1	NC_044945.1	ACGTTTCCTCGCAACGGATATGACTGGGACAACCAAACACC**T**TTAGAGGGCGCCGTTTACACG
NHV	NC_044943.1	ACGTTTCCTCGCAACGGATATGACTGGGACAACCAAACACC**T**TTAGAGGGCGCCGTTTACACG
L60	NC_044941.1	ACGTTTCCTCGCAACGGATATGACTGGGACAACCAAACACC**T**TTAGAGGGCGCCGTTTACACG
BA71V	U18466.2	ACGTTTCCTCGCAACGGATATGACTGGGACAACCAAACACC**T**TTAGAGGGCGCCGTTTACACG
E75	NC_044958.1	ACGTTTCCTCGCAACGGATATGACTGGGACAACCAAACACC**T**TTAGAGGGCGCCGTTTACACG
OURT 88/3	NC_044957.1	ACGTTTCCTCGCAACGGATATGACTGGGACAACCAAACACC**T**TTAGAGGGCGCCGTTTACACG
Benin 97/1	NC_044956.1	ACGTTTCCTCGCAACGGATATGACTGGGACAACCAAACACC**T**TTAGAGGGCGCCGTTTACACG
BEN/1/97	EF121428.1	ACGTTTCCTCGCAACGGATATGACTGGGACAACCAAACACC**T**TTAGAGGGCGCCGTTTACACG
Za	AY578708.1	ACGTTTCCTCGCAACGGATATGACTGGGACAACCAAACACC**T**TTAGAGGGCGCCGTTTACACG
Wb	AY578707.1	ACGTTTCCTCGCAACGGATATGACTGGGACAACCAAACACC**T**TTAGAGGGCGCCGTTTACACG
Wart	AY578706.1	ACGTTTCCTCGCAACGGATATGACTGGGACAACCAAACACC**T**TTAGAGGGCGCCGTTTACACG
Vic	AY578705.1	ACGTTTCCTCGCAACGGATATGACTGGGACAACCAAACACC**T**TTAGAGGGCGCCGTTTACACG
Ten	AY578704.1	ACGTTTCCTCGCAACGGATATGACTGGGACAACCAAACACC**T**TTAGAGGGCGCCGTTTACACG
Pr5	AY578703.1	ACGTTTCCTCGCAACGGATATGACTGGGACAACCAAACACC**T**TTAGAGGGCGCCGTTTACACG
Pr4	AY578702.1	ACGTTTCCTCGCAACGGATATGACTGGGACAACCAAACACC**T**TTAGAGGGCGCCGTTTACACG
o1	AY578701.1	ACGTTTCCTCGCAACGGATATGACTGGGACAACCAAACACC**T**TTAGAGGGCGCCGTTTACACG
Mk	AY578700.1	ACGTTTCCTCGCAACGGATATGACTGGGACAACCAAACACC**T**TTAGAGGGCGCCGTTTACACG
M1	AY578699.1	ACGTTTCCTCGCAACGGATATGACTGGGACAACCAAACACC**T**TTAGAGGGCGCCGTTTACACG
Ker	AY578697.1	ACGTTTCCTCGCAACGGATATGACTGGGACAACCAAACACC**T**TTAGAGGGCGCCGTTTACACG
K1	AY578696.1	ACGTTTCCTCGCAACGGATATGACTGGGACAACCAAACACC**T**TTAGAGGGCGCCGTTTACACG
F6	AY578694.1	ACGTTTCCTCGCAACGGATATGACTGGGACAACCAAACACC**T**TTAGAGGGCGCCGTTTACACG
E70	AY578692.1	ACGTTTCCTCGCAACGGATATGACTGGGACAACCAAACACC**T**TTAGAGGGCGCCGTTTACACG
cro3.5	AY578691.1	ACGTTTCCTCGCAACGGATATGACTGGGACAACCAAACACC**T**TTAGAGGGCGCCGTTTACACG
Cam	AY578689.1	ACGTTTCCTCGCAACGGATATGACTGGGACAACCAAACACC**T**TTAGAGGGCGCCGTTTACACG
Warthog	AY261366.1	ACGTTTCCTCGCAACGGATATGACTGGGACAACCAAACACC**T**TTAGAGGGCGCCGTTTACACG
Warmbaths	AY261365.1	ACGTTTCCTCGCAACGGATATGACTGGGACAACCAAACACC**T**TTAGAGGGCGCCGTTTACACG
Tengani 62	AY261364.1	ACGTTTCCTCGCAACGGATATGACTGGGACAACCAAACACC**T**TTAGAGGGCGCCGTTTACACG
Pretorisuskop/96/4	AY261363.1	ACGTTTCCTCGCAACGGATATGACTGGGACAACCAAACACC**T**TTAGAGGGCGCCGTTTACACG
Mkuzi 1979	AY261362.1	ACGTTTCCTCGCAACGGATATGACTGGGACAACCAAACACC**T**TTAGAGGGCGCCGTTTACACG
26544/OG10	NC_044947.1	ACGTTTCCTCGCAACGGATATGACTGGGACAACCAAACACC**T**TTAGAGGGCGCCGTTTACACG
R35	MH025920.1	ACGTTTCCTCGCAACGGATATGACTGGGACAACCAAACACC**T**TT**G**GAGGGCGCCGTTTACACG
N10	MH025919.1	ACGTTTCCTCGCAACGGATATGACTGGGACAACCAAACACC**T**TT**G**GAGGGCGCCGTTTACACG
Ken06.Bus	NC_044946.1	ACGTTTCCTCGCAACGGATATGACTGGGACAACCAAACACC**T**TT**G**GAGGGCGCCGTTTACACG
UgH03	EF121429.1	ACGTTTCCTCGCAACGGATATGACTGGGACAACCAAACACC**T**TT**G**GAGGGCGCCGTTTACACG
Kn	AY578698.1	ACGTTTCCTCGCAACGGATATGACTGGGACAACCAAACACC**T**TT**G**GAGGGCGCCGTTTACACG
RSA_2_2008	MN336500.1	ACGTTTCCTCGCAACGGATATGACTGGGACAACCAAACACC**TG**TAGAGGGCGCCGTTTACACG
CSFV	AF092448	No matches
PPV	AY583318.1	No matches
PRRSV	MH500776.1	No matches
PEDV	KY496315.1	No matches
PCV2	MK604485	No matches

*The left grey sequence is sense primer, the right grey sequence is anti-sense primer, and the middle grey sequence is MGB probe. Moreover, the bold letters indicate the mutated bases*.

### Construction of Standard Plasmid

A 1,941 bp complete fragment of ASFV B646L gene-encoded p72 protein and the ASFV B646L gene with EcoRI/XbaI restriction enzyme cutting site were obtained from the pUC57-p72 plasmid (synthesized by Sangon, Shanghai) by PCR using primers p72-Fwd: 5′-CGGAATTCATGGCATCAGGAGGAGC-3′ and p72-Rev: 5′-GCTCTAGATTAATGATGATGATGATGATGGGTACTG TAACG-3′. Then, the B646L gene was recombined with pFastBacI vector (Promega, USA) and transformed into DH5α (Takara, Dalian, China). The recombinant plasmid, pFastBacI-p72 plasmid, was extracted using Omega Plasmid mini kit (Omega, US). Restriction enzyme digestion and sequencing were used to determine whether the target fragment could be inserted correctly.

### Samples Preparation

The protocols of standard templates and clinical sample preparations were as follows. The concentration of the standard plasmid constructed in Section Construction of Standard Plasmid was detected using the NanoDrop One (ThermoFisher, US, AZY1812131) and diluted to the appropriate copy number, which began with 10^10^ copies/ml to 10-fold dilution. Copy number calculating formulas was shown as below.

$$\begin{array}{l} \text{Copy number }\left(copies/ml\right)\\ =\frac{6.02\times 10^{23}\left(copies/mol\right)\times C\left(g/ml\right)}{n\left(bp\right)\times \left(1.096\times 10^{-23}g/bp\right)\left(g/mol\right)} \end{array}$$

where C (g/ml) means the concentration of standard templates, and n (bp) means the genome size in base pairs.

Plasmids ranged from 10^10^ to 10^0^ copies/ml were as templates and positive controls for subsequent experiments. Inactivated clinical serum samples were obtained from the Henan Animal Husbandry Bureau and pig farms in Henan province, China. ASFV genomic DNA of clinical samples was extracted from swine serum samples by DNA Extraction Kit (Takara MiniBEST Viral DNA/RNA Extraction Kit, Takara, Dalian, China).

### Optimal Conditions of Quantitative PCR

An ABI 7500 Real-time PCR system (Applied Biosystems, USA) was used as a fluorescence quantification platform in this study. The reaction system was 10 μl, including 5 μl TaqMan Universal Master Mix II with uracil-N-glycosylase (purchased from Applied Biosystems, USA), 0.4 μl sense primer (ASFV-For), 0.4 μl anti-sense primer (ASFV-Back), 0.4 μl of probe, 1.8-μl nuclease-free water (Promega, USA), and 2 μl of template. The optimal concentrations of primers and the probe were then measured when the ASFV pFastBacI-p72 plasmid was 1 × 10^8^ copies/ml. Primers with optimal concentration were determined by 12.5, 25, 50, and 100 μM; meanwhile, the probe with optimal concentration was selected by 1.25, 2.5, 5, and 10 μM. The qPCR program was carried out as follows: initial denaturation at 95°C for 10 min, and at 95°C for 15 s, cycling 40 times, and at 60°C holding for 45 s. Negative and positive controls were set at the same time in a run.

### Digital PCR

QuantSudio^TM^ 3D Digital PCR System (ThermoScientific, US) was used as a cdPCR amplification platform. The volume of the reaction mixture was 20 μl, containing 10 μl 2× QuantSudio^TM^ 3D Digital PCR Master Mix (v2), 1.8 μl of each primer with optimal concentration determined by qPCR, 1.8 μl of the probe with optimal concentration determined by qPCR, 2.6 μl nuclease-free water (ThermoScientific, US), and 2 μl of DNA template. After sufficient mixed and briefly centrifuged, the 14.5 μl cdPCR reaction mixture was immediately loaded to the chips. Negative control and positive control were set for each test. Three replicates of the standard plasmid template were performed in each run. The program was in operation at 96°C for 10 min as a predenaturation step, at 60°C for 2 min, and at 98°C for 30 s, cycling 39 times, and finally, at 60°C for 2 min as a final elongation step.

### Limit of Detection for Chip Digital PCR

The limit of detection (LOD) for cdPCR was determined by the continuous dilution method. At the same time, the same templates were used for qPCR, approved by OIE (12) to compare the LOD between the two methods. The two amplification methods were repeated three times, and the data were analyzed statistically by logistic regression (Statistica 64, USA) (29).

### Specificity Analysis

In this analysis, the classical swine fever virus strain Shimen (AF092448), the porcine circovirus 2 strain HN-LB-16 (MK604485), the porcine reproductive and respiratory syndrome virus strain NADC30 (MH500776.1), and the porcine parvovirus strain China (AY583318.1) were kindly provided by Henan Agricultural University (Zhengzhou, Henan, China), and the porcine epidemic diarrhea virus strain CH/hubei/2016 (KY496315.1) was kindly provided by Jilin University (Changchun, Jilin, China). All these pathogens were detected by the nanofluidic cdPCR assay as nucleic acid templates.

### Repeatability Evaluation

The repeatability of cdPCR was evaluated by using the continuous dilution of ASFV standard plasmid containing 10^0^, 10^1^, 10^2^, 10^3^, and 10^4^ copies/ml as templates. On different days, three experiments were carried out, and each template in each experiment was repeated three times. The coefficient of variation (CV) was measured to analyze repeatability.

### Comparison of Chip Digital PCR With Quantitative PCR Approved by Office International des Epizooties and Commercial Kits

Comprehensive comparisons of cdPCR with qPCR approved by OIE and commercial kit (VetMAX™ African Swine Fever Virus Detection Kit, Thermofisher, US) were carried on by detecting 69 clinical samples. SPSS (version 21.0, IBM, USA) software and GraphPad Prism software (version 7.04; LA Jolla, California, USA) were used for statistical analysis, including the compliance rate, Bland and Altman analyses, and linear regression with the confidence limit of 95% (*P* < 0.05).

## Results

### Construction of Standard Plasmid and Identification of Target Gene

**Figure 1:**
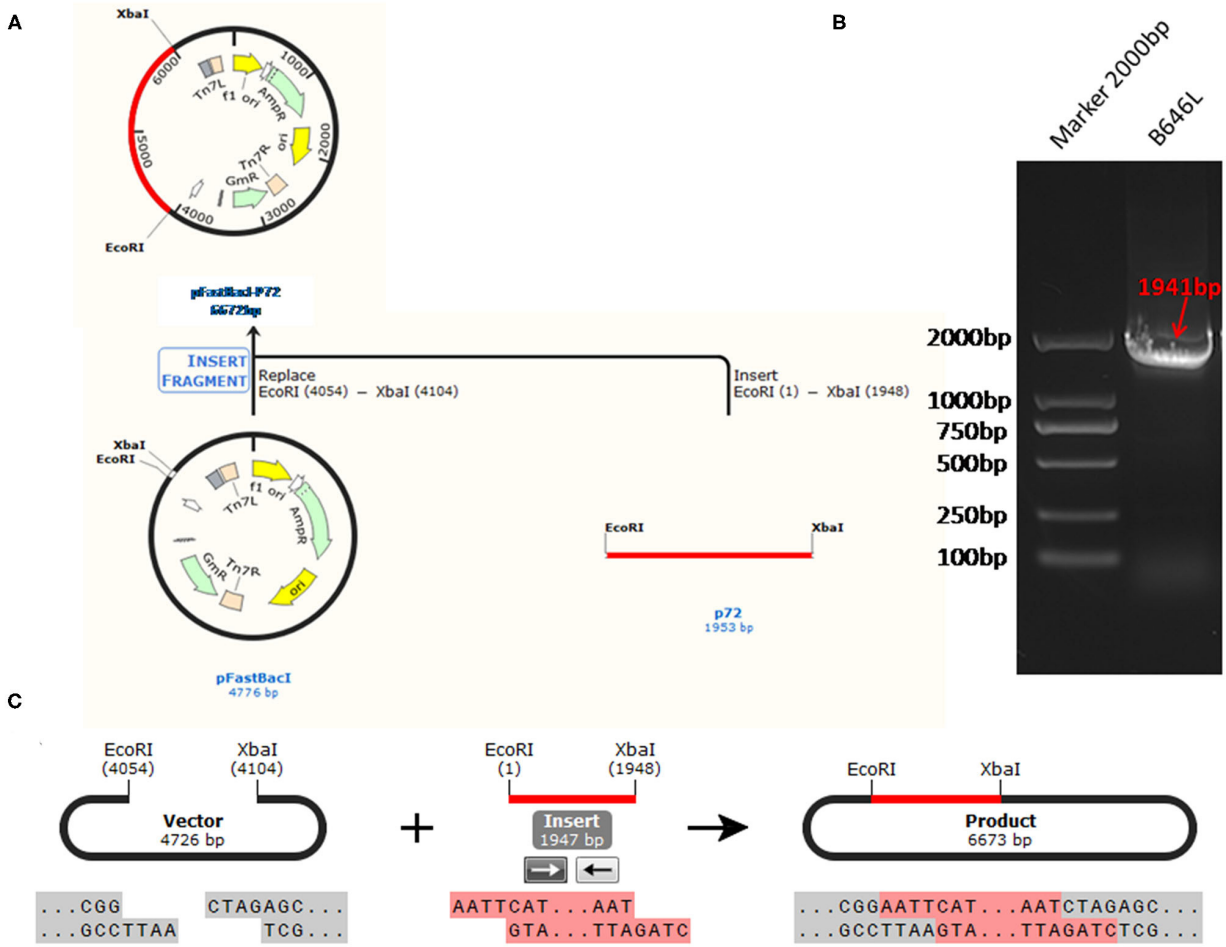
The strategy for the standard plasmid construction. **(A)** The recombination process of objective gene ASFV VP72 (B646L) and vector pFastBacI. **(B)** The size of target gene, ASFV B646L, was 1,941 bp on 1% agarose gel electrophoresis. **(C)** The cleavage sites of the recombination process is EcoR I/Xba I.

**Figure 2:**
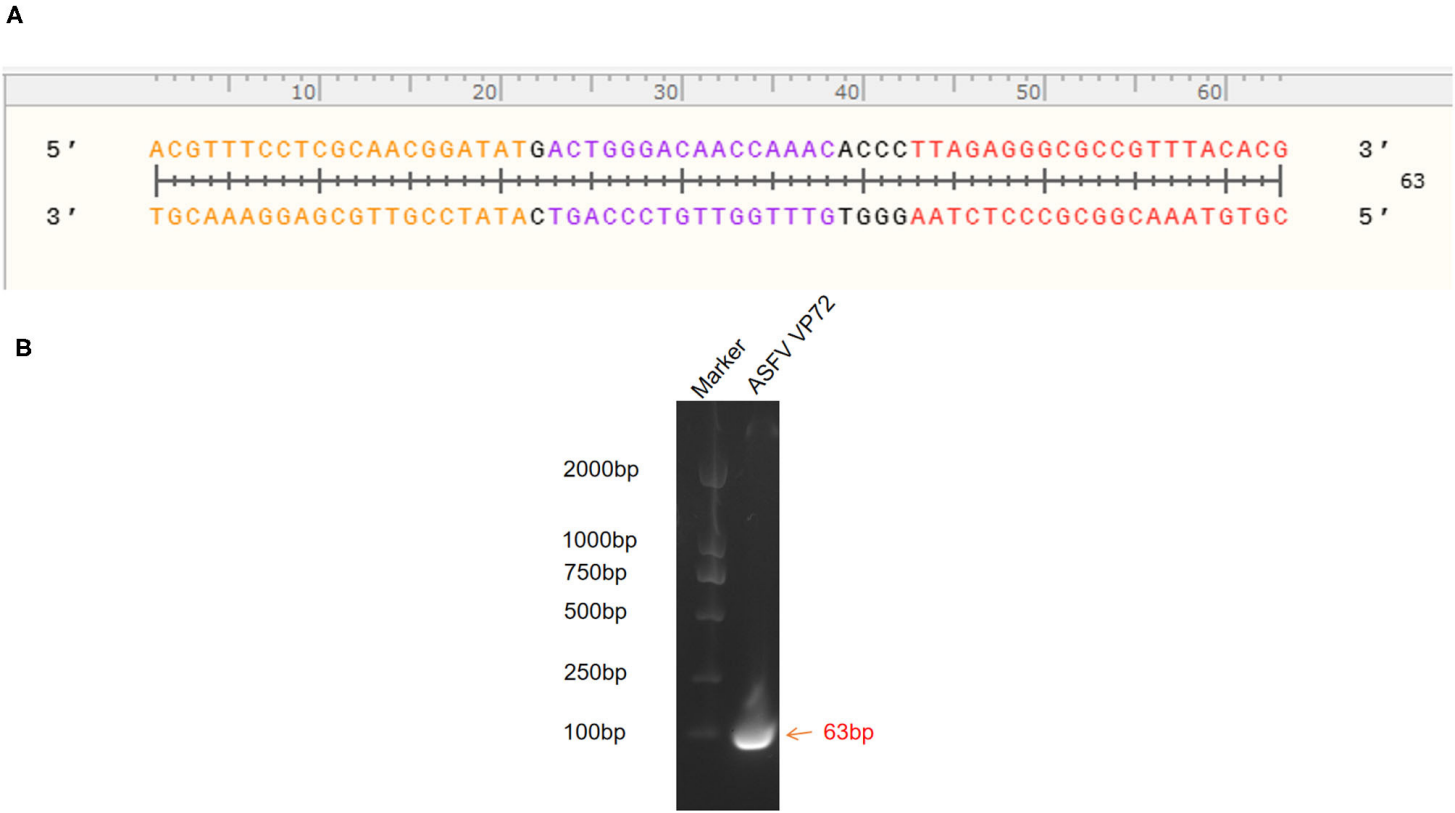
Identification of target gene. **(A)** Target nucleotide sequences of MGB probe and primers for cdPCR within the conserved region of ASFV B646L gene. Forward primer was marked in orange, reverse primer was marked in red and the probe was marked in purple. **(B)** Amplification products were analyzed by agarose gel electrophoresis.

The standard plasmid, pFastBacI-p72, was successfully constructed and identified by PCR and sequencing (Figure 1). The recombination process of objective gene ASFV p72 (B646L) and vector pFastBacI is shown in Figure 1A. The target gene, ASFV B646L, was amplified by PCR with 1,941 bp (Figure 1B) and spliced into two cleavage sites of restriction enzyme EcoRI and XbaI of vector pFastBacI. As shown in Figure 2A, double-stranded DNA sequences of the MGB probe and primers were marked in different colors within the conserved region of ASFV B646L. The size of the target gene amplified by cdPCR was ~63 bp. A single band of approximately 63 bp was obtained from PCR amplification products *via* 1% agarose gel electrophoresis (Figure 2B).

### Reaction Conditions of Quantitative PCR

**Figure 3:**
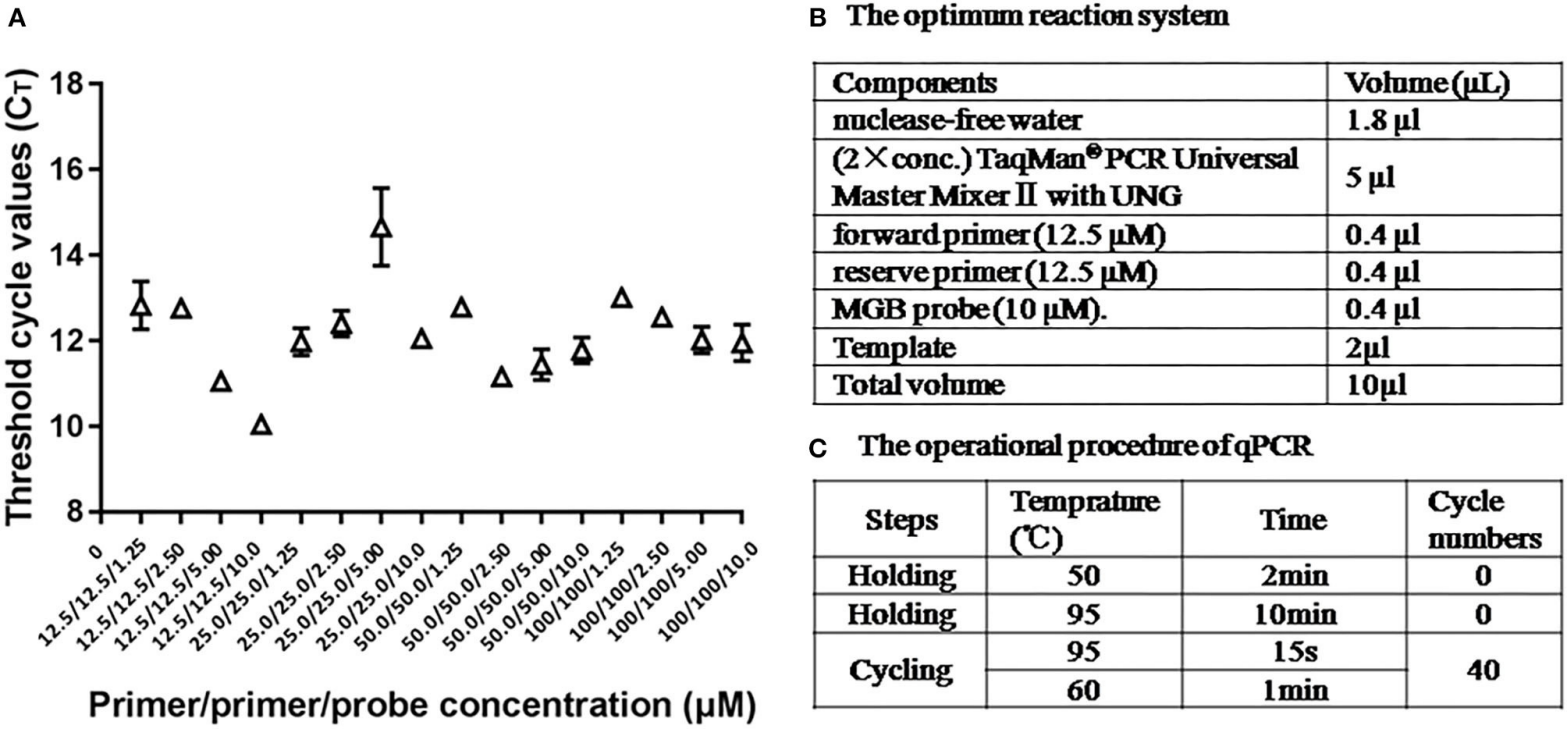
The optimum reaction condition for qPCR. **(A)** Influence of different concentrations PCR primers and probe with use of 108 copies/μl ASFV standard plasmid. **(B)** The optimum reaction system of qPCR. **(C)** The operational procedure of qPCR.

The optimum reaction condition for qPCR was detected *via* using a series of different concentrations of primers and the probe. The optimal concentration of primers was 12.5 μM, and the optimal concentration of the probe was 10 μM, at that time the C_T_ value was minimum (Figure 3A). The optimum reaction system and the program are shown in Figures 3B,C.

### Linear Standard Curve of Chip Digital PCR Assay

**Figure 4:**
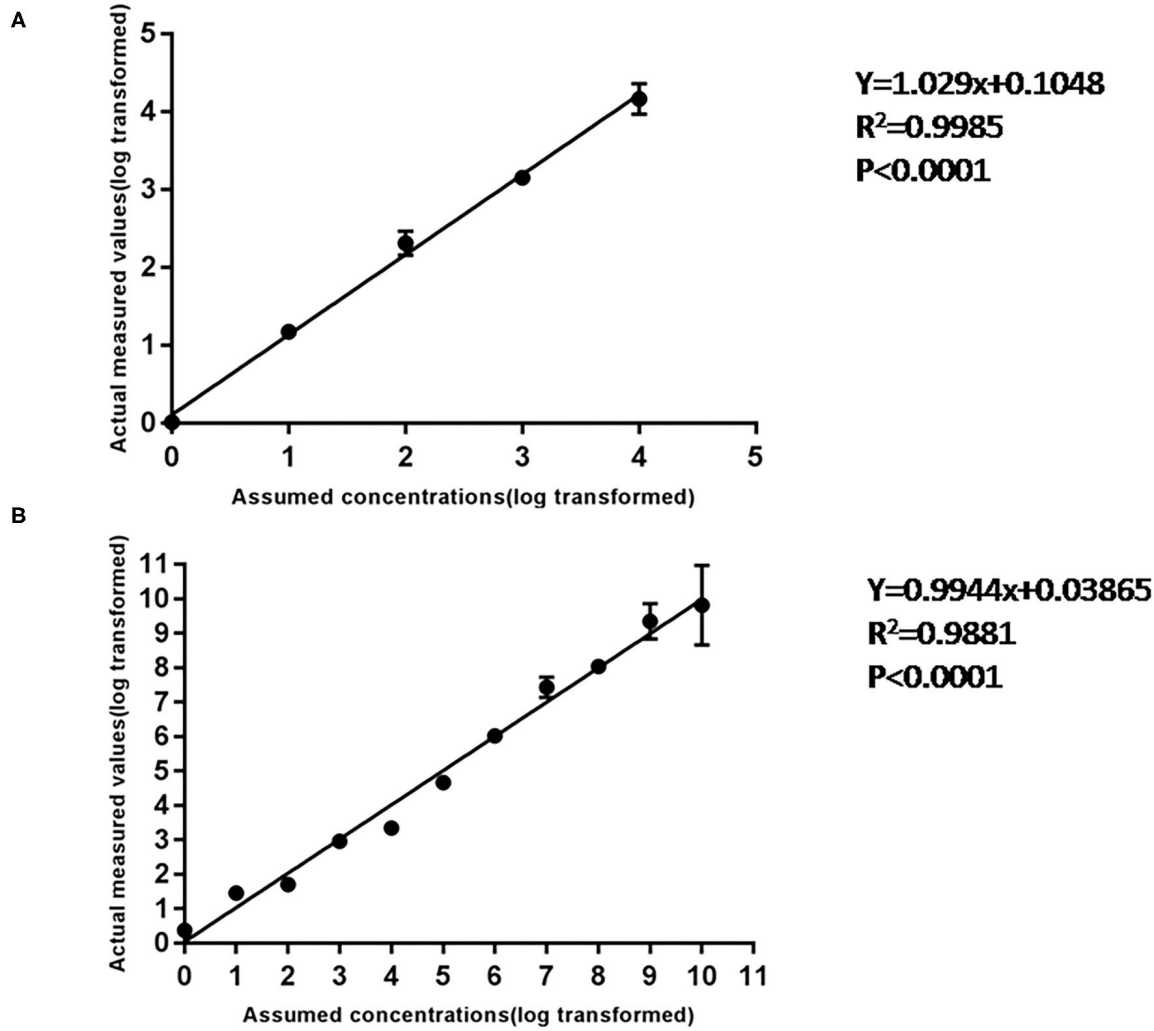
The standard curve of cdPCR and qPCR. **(A)** The standard curve of cdPCR. The slope of this linear fitting equation is 1.029 and the Y-intercept is 0.1048. **(B)** The standard curve of qPCR. The slope of this linear fitting equation is 0.9944 and the Y-intercept is 0.03865.

Using 10-fold diluted ASFV standard plasmid of 10^4^-10^−1^ copies/ml as templates, the standard curve of cdPCR was established. At the same time, the standard curve of qPCR confirmed by OIE was created by the same standard plasmid of 10^9^-10^0^ copies/ml. The trend line was highly linear with the assumed concentration for both cdPCR (Figure 4A) and qPCR (Figure 4B). The cdPCR assay proved greater linearity with an *R*^2^ of 0.9985 than the qPCR assay with an *R*^2^ of 0.9881 (Figure 4).

### Limit of Detection of Curve of Chip PCR Assay

**Figure 5:**
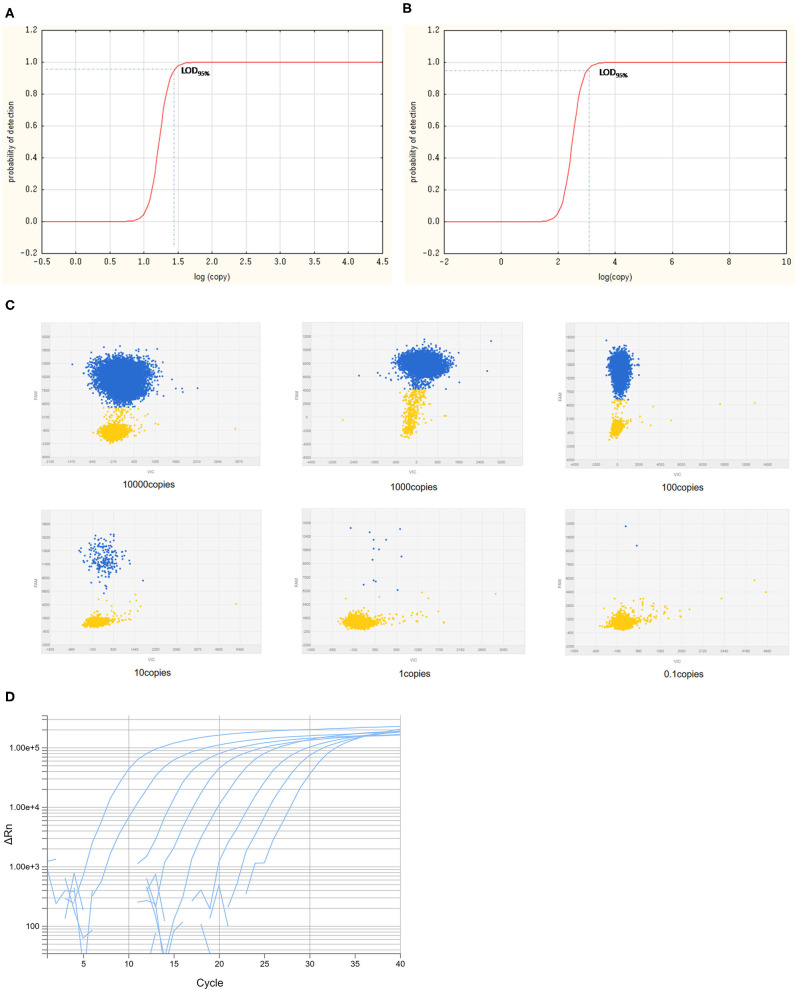
The LOD_95%_ of cdPCR and qPCR assay. Logit analysis plots of the cdPCR **(A)** and qPCR **(B)** used in the study show the LOD_95%_, which are the minimum amounts of DNA detectable with a 95% probably. The amplification curve of cdPCR **(C)** and qPCR **(D)** is obtained with 10-fold diluted standard plasmids.

The LODs for both cdPCR and qPCR approved by OIE were determined using the same set of primers and the probe with ASFV standard plasmid diluted 10 times as templates. The results are shown in Figure 5. Using the least-squares modeling approach and logistic regression analysis, the LOD_95%_ of the cdPCR assay was 1.48 Log10 copies per reaction, that is, 30.1995 copies per reaction (Figures 5A,C), and the LOD_95%_ of the qPCR assay was estimated as three Log10 copies per reaction, that is, 1,000 copies per reaction (Figures 5B,D). Hence, the LOD_95%_ of cdPCR assay was approximately 33 times higher than that of the qPCR assay. The cdPCR assay was more sensitive than the qPCR assay.

### Specificity Analysis

**Figure 6:**
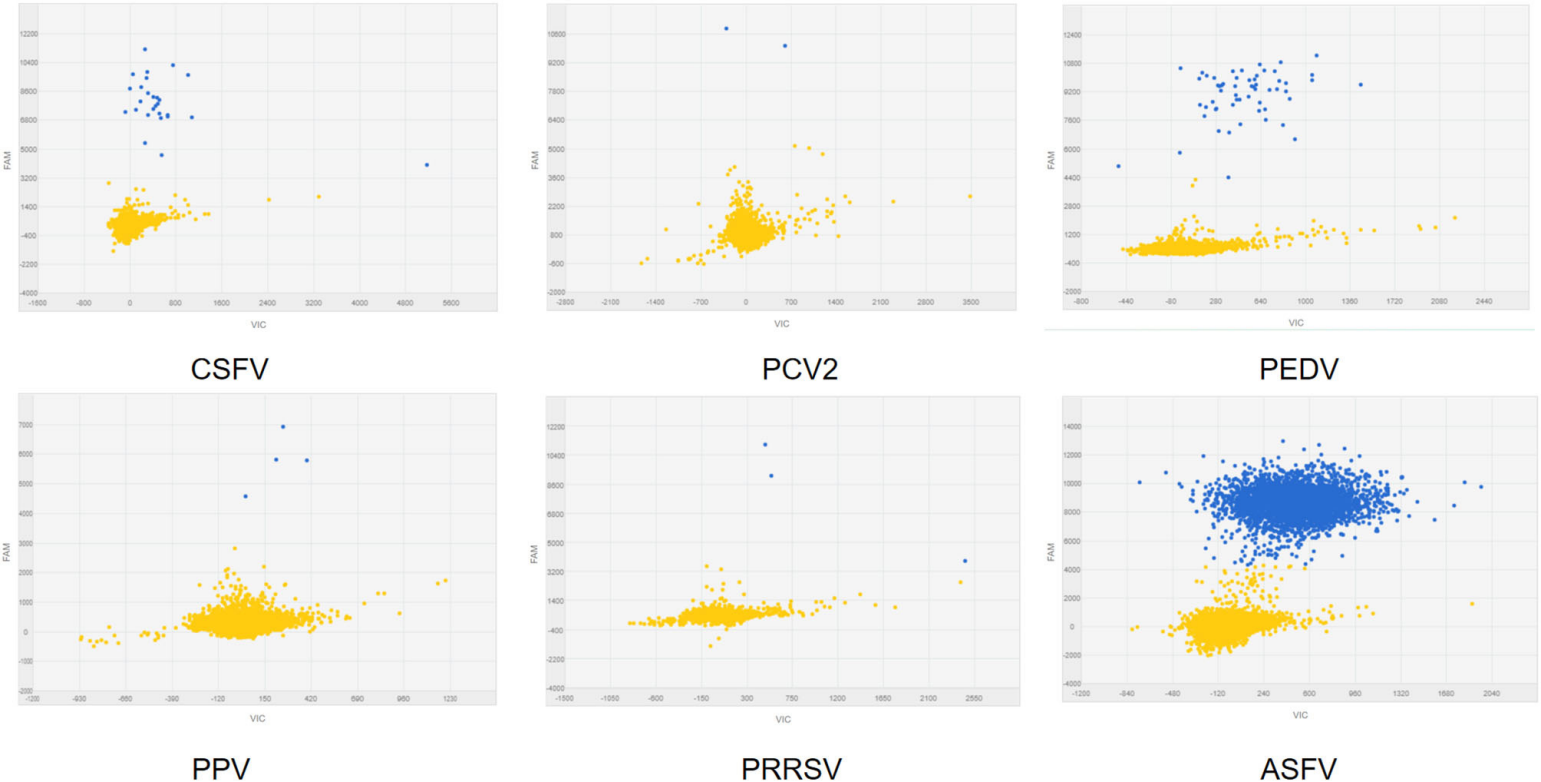
The specificity of cdPCR assay. Only the ASFV pFastBacI-p72 standard plasmid was positive, i.e., nucleic acid templates of other pathogens were negative.

To analyze the specificity of cdPCR, DNA and complementary DNA, extracted from other swine viruses containing classical swine fever virus, porcine parvovirus, porcine circovirus 2, porcine reproductive and respiratory syndrome virus, and porcine epidemic diarrhea virus, were used as templates, and ASFV pFastBacI-p72 standard plasmid was used as a positive control in specificity assay. The standard plasmid was positive, but nucleic acid templates of the other five pathogens were negative (Figure 6 and Table 2). The result was strongly in line with our theorized expectations that the sequences of primers and probe for the ASFV cdPCR did not match with the nucleic acid sequences of any other swine pathogens (Table 1). All results mentioned earlier demonstrated that the ASFV cdPCR detection method had good specificity.

**Table 2:** Concentration of CSFV, PPV, PCV2, PRRSV, and PEDV, and ASFV standard plasmid by cdPCR assay.

**Isolation virus**	**Mean concentration (copies/μl)**
CSFV	1.027
PPV	0.616
PRRSV	0.532
PEDV	1.391
PCV2	0.692
ASFV	845.11

### Repeatability Analysis

**Figure 7:**
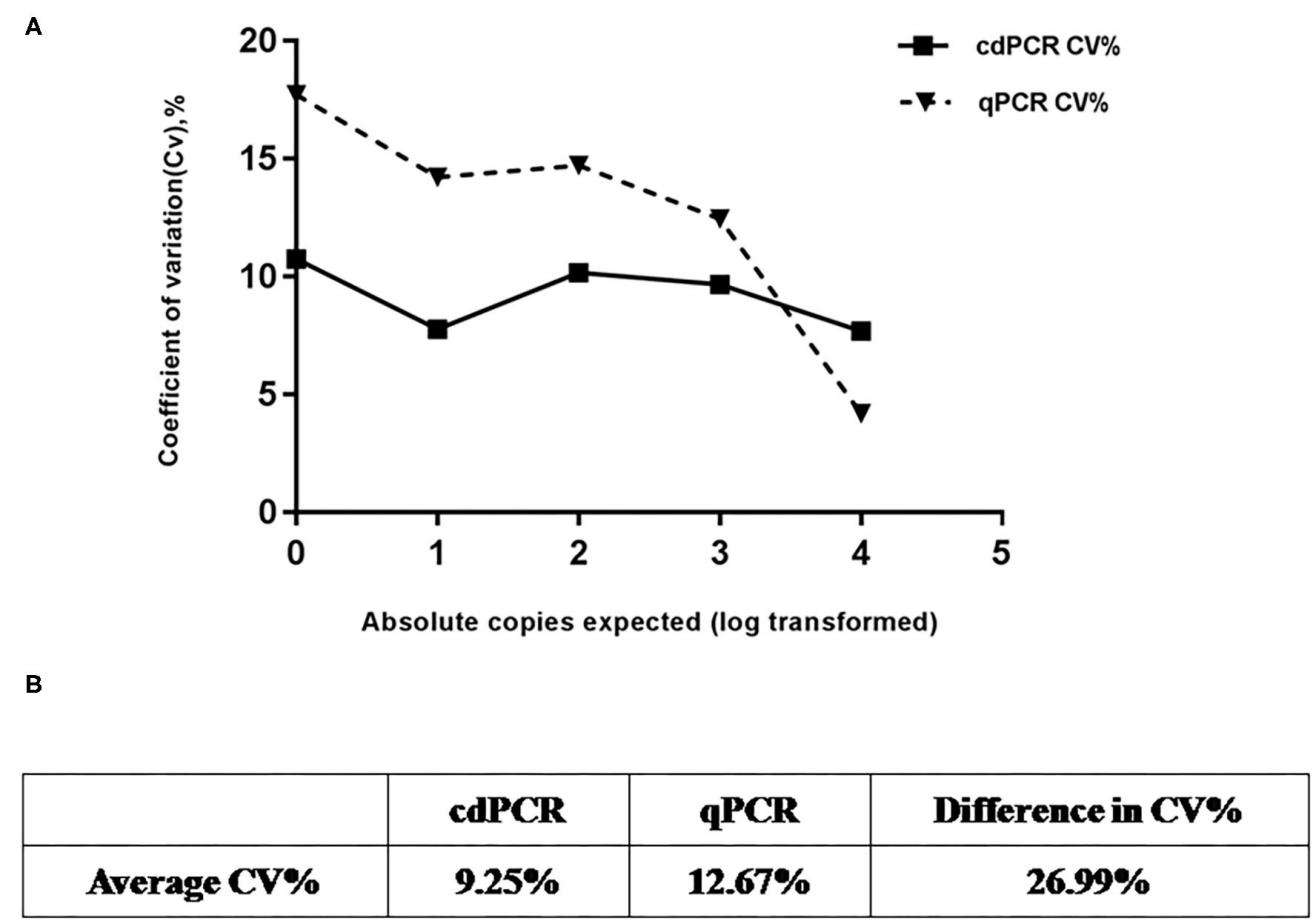
Repeatability analysis. **(A)** Trend line comparing coefficient of variation (CV%) for cdPCR and qPCR at different concentration points. The square with the full line stands for CV % of cdPCR and the triangle with the dotted line indicated as the CV% of qPCR. **(B)** Table shows that average CV% value of cdPCR (9.25%) is lower than that of qPCR (12.67%).

Using serially diluted standard plasmids as templates for cdPCR amplification, three independent experiments were performed by different operators at different times. The cdPCR assay displayed good repeatability and a low coefficient of variation between most dilution points (Figure 7). The cdPCR assay had an average CV% of 9.56%, which was lower than the average CV of 12.67% of qPCR approved by OIE, resulting in an average decrease in CV% of 26.99% (Figure 7).

### Analysis With Clinical Samples

To calculate the coincidence rate of the cdPCR method to detect ASFV, we compared, respectively, the cdPCR method established in this study with the qPCR approved by OIE and commercial qPCR kit (VetMAX™ African Swine Fever Virus Detection Kit, Thermofisher, US), by testing 69 swine serum samples.

**Figure 8:**
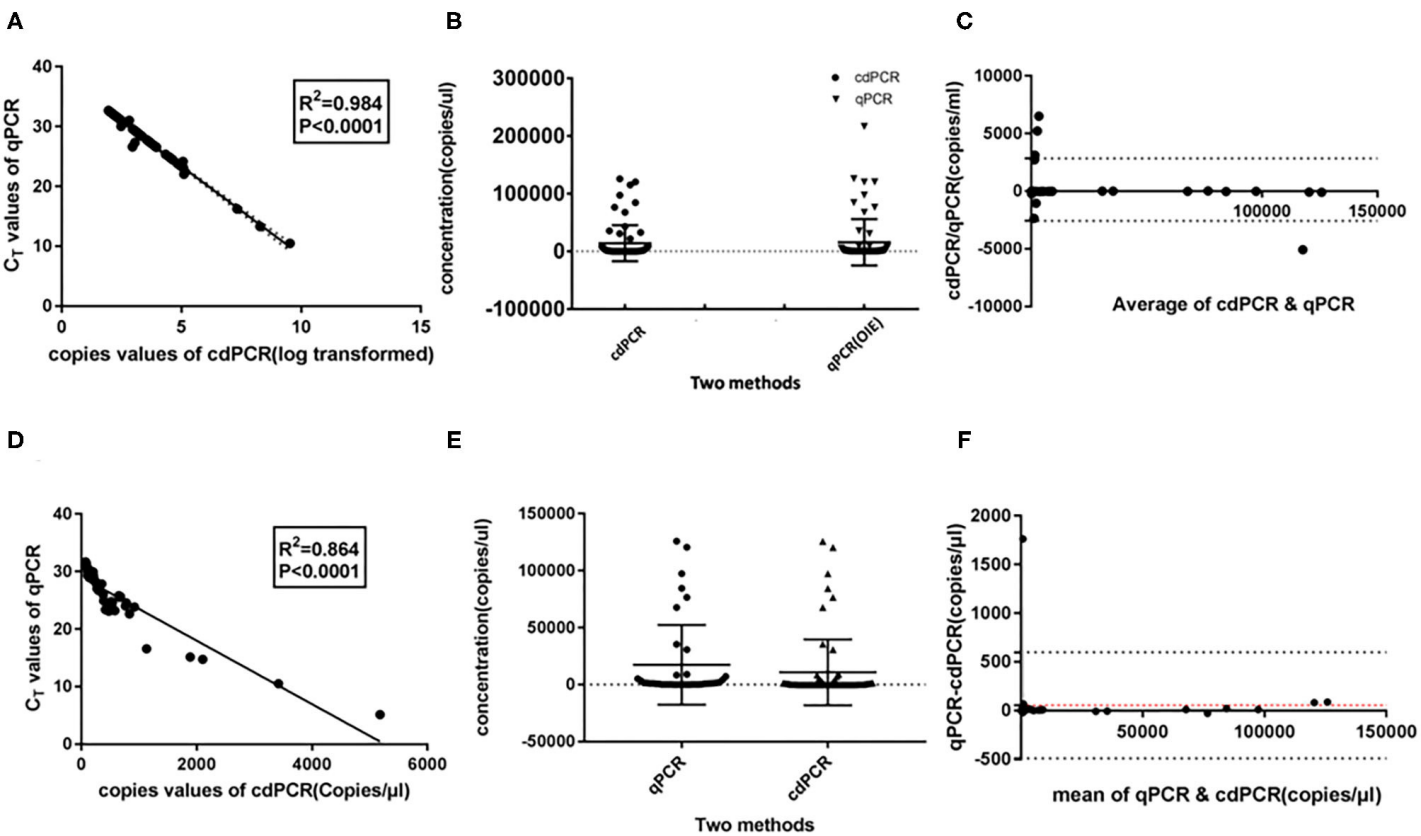
Statistical analysis for cdPCR in testing clinical blood samples. Pearson correlation and linear regression analysis showed well-consistency between qPCR approved by OIE (*R*^2^ = 0.864) **(A)** as well as between cdPCR and commercial qPCR kit (*R*^2^ = 0.864) **(D)**. The standard deviation of cdPCR is lower than that of qPCR approved by OIE **(B)** and that of commercial qPCR kit **(E)**. **(C)** Bland and Altman analyses plots for cdPCR and qPCR approved by OIE demonstrated that 5.797% (4/69) dots were outside the region between 95% lower limit of agreement and 95% upper limit of agreement, and the bias value for this agreement's range was 1,381 copies/ml (*P* < 0.05) by Graphpad Prism 7.04. **(F)** Bland and Altman analyses plots for cdPCR and the kit demonstrated that 1.449% (1/69) dots were outside the region between 95% lower limit of agreement and 95% upper limit of agreement with that of the bias value.

As shown in Table 3A, the cdPCR and qPCR approved by OIE have, respectively, detected 50 and 48 positive samples in the clinical diagnosis of 69 domestic pigs. The sensitivity of the cdPCR assay was 95.83% (46/48), and the specificity was 94.44% (17/21). The positive coincidence rate of the cdPCR assay was 92% (46/50). The total coincidence rate of the two methods was 91.30% (63/69), and the kappa value reached 0.789 (*P* < 0.0001). There was significant consistency between the two from the results. Furthermore, quantitation of the correlation between the two was analyzed by Pearson correlation and linear regression analysis on 46 positive samples (Figure 8). The quantitative analysis of the correlation between the two showed that they had a good correlation because the *R*^2^ value of linear regression was 0.984 (*P* < 0.0001) (Figure 8A). The standard deviation of cdPCR was lower than that of qPCR by Mann–Whitney *U* test (Figure 8B). Bland and Altman analyses plots (Figure 8C) demonstrated that 5.797% (4/69) dots were outside the region between 95% lower limit of agreement and 95% upper limit of agreement, and the bias value for this agreement's range was 1,381 copies/ml (*P* < 0.05) by Graphpad Prism 7.04.

**Table 3A:** Testing of clinical samples by cdPCR and qPCR assay approved by OIE.

**Samples**	**qPCR approved by OIE**			**Summary**
		**Positive**	**Negative**	
cdPCR	Positive	46	4	50
	Negative	2	17	19
Summary		48	21	69

The data in Table 3B show that 45 of 69 samples were judged to be positive by VetMAX™ African Swine Fever Virus Detection Kit. The sensitivity of the cdPCR assay was 86.27% (44/50), and the specificity was 94.44% (17/18). Furthermore, the positive coincidence rate and the overall coincidence rate of the cdPCR assay were 88% (44/50) and 89.86% (62/69), respectively, and the kappa value was 0.800 (*P* < 0.0001). Those seven samples with inconsistent results between two assays were tested with cdPCR three times to exclude false-positive events, and negative and positive controls were included in all trials. All seven samples were declared as positive samples by cdPCR tests. In addition, the quantitative agreements were evaluated using Pearson correlation and linear regression analysis on 44 positive samples. The quantitative analysis of the correlation between the two showed that they had a good correlation because the *R*^2^ value of linear regression was 0.864 (*P* < 0.0001) (Figure 8D). The standard deviation of cdPCR was lower than that of the kit (Figure 8E). Bland and Altman analyses plots (Figure 8F) demonstrated that 1.449% (1/69) dots were outside the region between 95% lower limit of agreement and 95% upper limit of agreement with that of the bias value. Within the consistency limit, the absolute value of the difference between the concentration of the sample to be measured by cdPCR and qPCR was 1,762.59 copies/ml (the top point in Figure 8F), and the average value of the difference was 54.85 copies/ml determined by Graphpad Prism 7.04.

**Table 3B:** Testing of clinical samples by cdPCR and commercial kit.

**Samples**	**qPCR (commercial kit)**			**Summary**
		**Positive**	**Negative**	
cdPCR	Positive	44	6	50
	Negative	1	18	19
Summary		45	24	69

Above all, the cdPCR technology developed in this study had comparable performances with the qPCR approved by OIE as well as VetMAX™ African Swine Fever Virus Detection Kit in terms of detecting ASFV clinical samples.

## Discussion

ASFV has been widely spreading outside Africa to Europe (30, 31) and most recently to Georgia (32), China (33), Cambodia (12), South America (21, 34, 35), and so on, even to reach almost every corner of the world, which is a significant transboundary and emerging virus (36, 37). ASF is a serious and highly contagious disease with high mortality, causing acute hemorrhagic fever in domestic pigs and wild boars (38–40). Hence, ASF was the biggest threat to the world pork industry (41). Although vaccination is the preferred method for controlling the disease, the development of safe vaccines to protect pigs from ASFV has not achieved significant success since the first isolation of ASFV (42). Because there are no safe and efficacious vaccines, the key of current surveillance and control measures against ASF is firstly to cut off the transmission of the pathogen once ASFV clinical symptoms are observed. Then, diagnosis and confirmation of ASFV require laboratory testing. The traditional method of diagnosing ASFV is using qPCR to measure the ASFV genomic DNA. However, the quality of the standard curve affects the accuracy quantification of qPCR. If the standard curve is unstable, ASFV DNA quantification will be inexact (43). Additionally, CT values in qPCR related to amplification efficiencies are obtained from the amplification of standards and the samples. Also, several factors, such as inhibitors, amount of total DNA, and variations between the primers and the probe, may cause the false amplification of the templates, resulting in the CT values going up. Digital PCR as a novel approach to nucleic acid quantification has been used in several aspects with equal or superior performance to qPCR.

Digital PCR can realize an absolute target quantification without standards and the standard curve. Nanofluidic cdPCR running on QuantStudio 3D digital PCR platform (Applied Biosystems) has been applied as a useful tool for sensitive and accurate detection of norovirus low-copy targets (28), quantification of bacterial pathogens (44), quantifying microRNAs in infarction patients (45), and detection of enterotoxigenic *Bacteroides fragilis* (46). Although droplet digital PCR has been reported being applied to detecting ASFV (47), in this paper, we applied nanofluidic cdPCR on QuantStudio 3D digital PCR platform to diagnose ASF for the first time and assess the applicability of detection ASFV by using cdPCR on aspects such as sensitivity, specificity, reproducibility, among others.

The 53 complete ASFV genome sequences in the GenBank database were aligned, and a suite of primers and an MGB probe were designed based on a highly conserved fragment of the B646L gene coded p72 protein. Various properties of cdPCR assays, such as sensitivity, repeatability, and coincidence rate, were evaluated after optimizing reaction conditions. The linearity analysis of cdPCR detection was performed using 10-fold diluted ASFV standard plasmid as templates, with initial concentration of 10^4^ to 10^−1^ copies/ml. The results showed that the limit detection of cdPCR [30.1995 copies per reaction (*n* = 3)] was approximately 33 times higher than that of qPCR approved by OIE (1,000 copies per reaction) (12). Also, the limit detection of cdPCR did correlate well with that of an improved new real-time PCR assay established by Tignon et al. (5.7–57 copies per reaction) (21). The sensitivity of the cdPCR detection method has been significantly improved.

The statistics offer further support in that cdPCR is a perfect tool to detect ASFV. Detecting 69 inactivated clinical serum samples by cdPCR and other techniques showed good consistency with cdPCR and qPCR approved by OIE as well as VetMAX™ African Swine Fever Virus Detection Kit (Thermofisher, US). The positive detection rate of the cdPCR method established in this study was 72.46% (50/69), which had a better performance than both qPCR approved by OIE [69.57% (48/69)] and VetMAX™ African Swine Fever Virus Detection Kit [65.22% (45/69)]. Additionally, the cdPCR assay did not react with other swine viruses. Both Bland and Altman analyses and line regression analysis exhibited that cdPCR carried out comparably better than the other two methods.

There are some limitations of the novel cdPCR. That specific equipment is required for nanofluidic cdPCR, which makes it hard to popularize and be widely applied. A specialized nanofluidic chip that accompanies QuantStudio 3D digital PCR platform is a little bit expensive. So, qPCR assay is more economical than cdPCR. Also, cdPCR can only amplify a maximum of 24 samples in a single run, 72 samples fewer than qPCR for a single run. Although this shortcoming of cdPCR can be overcome by adding the number of the ProFlex™ 2× Flat PCR System or Dual Flat Block GeneAmp™ PCR System (Applied Biosystem, US), the cost is too high. Therefore, qPCR is more applicable in detecting large numbers of clinical samples than cdPCR. However, cdPCR is suitable for the quantification of low copy numbers, especially when the laboratory standard of quantification qPCR for virus genomic DNA/RNA is limited (44). Taken together, the method of using cdPCR to detect ASFV in serum samples has been established and feasible. The cdPCR, as a good tool, can be applied to the absolute quantification of ASFV.

## Data Availability Statement

The raw data supporting the conclusions of this article will be made available by the authors, without undue reservation.

## Ethics Statement

Ethical statement is not applicable because sample collection has been gathered. Written informed consent was obtained from the owners for the participation of their animals in this study.

## Author Contributions

AW contributed to study design, laboratory supervision, and manuscript editing. RJ contributed to study design, doing experiments, data analysis and manuscript drafting, editing, and writing. HL contributed significantly to the collection of laboratory data. YC, JZ, YW, and WZ contributed to laboratory quality control and data collection. YL polished the language of the manuscript. PD helped perform the analysis with constructive discussions. GZ and AW contributed to study design, laboratory supervision, and manuscript editing. All authors contributed to the article and approved the submitted version.

## Conflict of Interest

HL, YC, JZ, YL, YW, and WZ were employed by Henan Zhongze Biological Engineering Co. Ltd. The remaining authors declare that the research was conducted in the absence of any commercial or financial relationships that could be constructed as a potential conflict of interest.
